# Prevalence of High HDL Cholesterol and Its Associated Factors Among Tunisian Women of Childbearing Age: A Cross-Sectional Study

**DOI:** 10.3390/ijerph18105461

**Published:** 2021-05-20

**Authors:** Fatma Ben Cherifa, Jalila El Ati, Radhouene Doggui, Myriam El Ati-Hellal, Pierre Traissac

**Affiliations:** 1 SURVEN (Nutrition Surveillance and Epidemiology in Tunisia) Research Laboratory, INNTA (National Institute of Nutrition and Food Technology), 11 Rue Jebel Lakhdar, bab Saadoun, 1007 Tunis, Tunisia; fatmabencherifa@yahoo.fr (F.B.C.); jalila.elati@yahoo.fr (J.E.A.); doggui.radhouene@gmail.com (R.D.); 2 Laboratory Materials Molecules and Applications, IPEST (Preparatory Institute for Scientific and Technical Studies), University of Carthage, P.B. 51, 2070 Tunis, Tunisia; 3 MoISA-Univ Montpellier, CIRAD, CIHEAM-IAMM, INRAE, Institut Agro, IRD, 911 Av. Agropolis, 34394 Montpellier, France; pierre.traissac@ird.fr

**Keywords:** high HDL-C, prevalence, risk factors, Tunisian women of childbearing age

## Abstract

The protective role of high high-density lipoprotein cholesterol (HDL-C) against cardiovascular risk has been questioned recently. Due to the increasing trend of cardiovascular diseases (CVD) in Tunisia, this study aimed to determine the prevalence of high HDL-C and its associated factors in Tunisian women of childbearing age. A cross-sectional survey was conducted among a subsample of 1689 women, aged 20 to 49 years, in the Great Tunis region. Data on socio-demographic and lifestyle factors were collected by a questionnaire. Overall adiposity was assessed by body mass index (BMI). All biological variables were assayed in blood samples coated with anticoagulant ethylene diamine tetra acetic acid (EDTA) by enzymatic methods. Stata software (2015) was used for data management and statistical analysis. High HDL-C values were recorded in 26.6% of selected women. After adjustment for all socio-demographic and lifestyle factors, age, hypertension, and smoking were negatively associated with high HDL-C levels, while family history of cancer was positively associated with high HDL-C in women. An additional investigation on the relationship between high HDL-C and cancer risk should be performed due to controversial results.

## 1. Introduction

Cardiovascular diseases (CVDs) are the leading cause of deaths worldwide [1]. In Tunisia, a Middle East and North African (MENA) country with eleven million inhabitants, CVDs are responsible of 23.9% and 28.7% of deaths for men and women, respectively [2]. Several epidemiological studies have shown an inverse and independent association between high-density lipoprotein cholesterol (HDL-C) and CVDs [3,4]. The protective effect of HDL-C is mainly due to its transport of excess cholesterol from peripheral tissues to the liver. This pathway is called the reverse cholesterol transport system (RCT) [5,6,7]. Additional protective properties of HDL-C include its antioxidant, anti-inflammatory, anti-infectious, and anti-thrombotic potential [8,9,10]. Recently, the prognostic importance of HDL-C as a specific risk factor for CVDs has been questioned, since many therapies attempting to increase HDL-C failed to improve clinical outcomes [10,11]. Moreover, other studies reported that extremely high levels of HDL-C are associated with high mortality risk [12,13,14].

Due to the increasing trend of CVDs in Tunisia, this study aimed to estimate the prevalence of HDL-C and investigate the associations between high HDL-C levels and socio-demographic, metabolic, and lifestyle factors in Tunisian women of childbearing age.

## 2. Materials and Methods

### 2.1. Sampling and Study Population

A cross-sectional survey was carried out between March 2009 and January 2010 in the Greater Tunis region, a mainly urban area around the capital city (2.5 million inhabitants, of whom 92% live in urban areas and 8% in rural areas). Sampling was carried out by the National Institute of Statistics according to a stratified random survey in two stages. Totally, 76 districts were selected first according to the governorate of residence, then according to the environment (urban and rural). From each district, 20 households were randomly selected, and all persons aged six months to 49 years were included. In the present study, a subsample of non-pregnant women aged 20 to 49 years old was used.

### 2.2. Socioeconomic and Demographic Variables

Data on the woman’s age, marital status, parity, menopause, level of education, lifestyle: smoking (yes = current use of any tobacco products that are either sniffed, sucked, or chewed, e.g. cigarettes, pipes, cigars, and shisha, no = no tobacco use), alcoholism (yes = consumption of an alcoholic drink at least once a year, no = never or less than once a year), sport activity (yes = doing a specific physical activity on a regular basis, no = no practice of any physical activity on a regular basis), occupation, household size, and professional activity (yes = being professionally active, no = unemployed) were collected by a questionnaire. An economic level score for the household was calculated from six variables describing the dwelling and eleven variables coding household ownership of appliances. The total score obtained per household was coded in terciles corresponding to low, medium, or high economic level [15]. Family history for chronic non-communicable diseases was collected through a specific question: “Do you have a family history for cardiovascular diseases, hypertension, cancers, diabetes, and obesity?”

### 2.3. Anthropometric Variables

Measurements of height, weight, and waist circumference were performed according to standardized procedures [16]. Height was measured to the nearest 0.1 cm with a stadiometer. Body weight was measured to the nearest 0.1 kg. Waist circumference (WC) was measured to the nearest 0.1 cm using a metric fiberglass tape. Overall adiposity was assessed by BMI (weight (kg)/height^2^ (m^2^)). BMI was categorized as underweight < 18.5 kg/m^2^, overweight ≥ 25 kg/m^2^, and obese ≥ 30 kg/m^2^.

### 2.4. Biological Variables

#### 2.4.1. Analysis

Five ml blood samples were collected in tubes coated with the anticoagulant ethylene diamine tetra acetic acid (EDTA). All samples were kept at 4–5 °C and sent the same day to the Clinical Biology Laboratory of the National Institute of Nutrition and Food Technology, then centrifuged at 4000× *g* for 10 min and stored at −20 °C until analysis. Blood pressure (BP) was measured at rest twice and at a time interval of at least 15 min using a BP monitor. Fasting blood glucose, total cholesterol (TC), triglyceridemia, high-density lipoprotein cholesterolemia (HDL-C), low-density lipoprotein cholesterolemia (LDL-C), and apoliproteins A-I (ApoA-I) and B (ApoB) were assayed by enzymatic methods on a Synchron analyzer and calibrator using Beckman reagents. The accuracy was evaluated by quality control samples (BioRad, Hercules, CA, USA).

#### 2.4.2. Threshold Values

Hypertension was defined as having an average systolic blood pressure (SBP) ≥ 140 mmHg and/or diastolic blood pressure (DBP) ≥ 90 mmHg or taking medication for high BP [17]. Diabetes mellitus was defined as a fasting glucose level ≥ 126 mg/dL (7 mmol/L) and/or the use of antidiabetic treatment [18]. An HDL-C level of < 50 mg/dL in women was considered low, while an HDL-C level of ≥60 mg/dL was considered high [19]. Metabolic syndrome was present in the case of women at central obesity (WC > 80 cm) and at least two of the following risk factors: SBP ≥ 130 mmHg or DBP ≥ 85 mmHg or antihypertensive treatment; glucose ≥ 1 g/L (5.6 mmol/dL) or diagnosis of type 2 diabetes mellitus; and triglyceridemia ≥ 1.5 g/L (1.7 mmol/L) or treatment of high triglyceridemia [20]. Other CVD risk factors were obtained with ratios of TC/HDL-C and Apo-B/Apo-A1 higher than 4.5 and 1, respectively [21,22].

### 2.5. Data Management and Statistical Analysis

Data entry, including quality checks and validation by double entry, was performed with EpiData Software version 3.1 (The Epidata Association, Odense, Denmark) [23]. Stata software (StataCorp, College station, TX, USA) [24] was used for data management and statistical analysis. Results are shown as the mean ± standard error. The association between categorical variables was evaluated by the chi-squared test. The association of high HDL-C with the different cofactors was assessed by operating a multivariable logistic regression after the selection of an appropriate reference category. Adjustment was done for age, socio-demographic variables (education level, marital status, professional activity, household economic level, household size, and living area), lifestyle (self-reported practice of regular physical activity and smoking), and biological factors (hypertension status, diabetes status, family history of cancer, and family history of cardiovascular diseases). The Wald test was used for regression coefficient comparison. For tests and confidence intervals, an alpha threshold of 5% was chosen.

## 3. Results

### 3.1. General Characteristics of the Subjects

The survey was conducted among 1689 women aged 20 to 49 years (average age = 36.1 ± 0.3 years), of which 67.7% were married and 32.2% were single, separated, divorced, or widowed at the time of the survey. The majority of women (40.2%) were multiparous, with three or more children, while 26.9% had one or two children. Only 10.9% of women had never attended school, 53.2% had reached the secondary or university level, and 32.8% reported working outside.

### 3.2. Characteristics of Women According to HDL-C Levels

The average HDL-C concentration in Tunisian women of childbearing age was 1.36 ± 0.02 mmol/L (52.6 ± 0.8 mg/dL). High HDL-C values were recorded in 26.6% of subjects, while 14.3% were with low HDL-C concentrations. When adjusted by age, the prevalence was, respectively, 26.6% (95% CI: 22.2–31.4) and 14.7% (11.5–18.6). Table 1 displays the characteristics of the selected participants according to HDL-C levels. Age as well as area of living, menopause, professional activity, smoking, drinking alcohol, sport activity, diabetes, metabolic syndrome, lipid-lowering treatment, family history of CVDs, family history of hypertension, family history of diabetes, family history of obesity, fasting blood glucose, and LDL-C had no effects on HDL-C concentrations. However, marital status, parity, economic level, overweightness, obesity, abdominal obesity, hypertension, family history of cancer, TC, triglyceridemia, TC/HDL-C ratio, ApoA-I, ApoB, ApoA-I/ApoB ratio, SBP, and DBP were significantly associated with HDL-C values.

### 3.3. Individual Association between Socio-Demographic, Lifestyle, and Biological Characteristics with a High HDL-C Level

**Figure 1:**
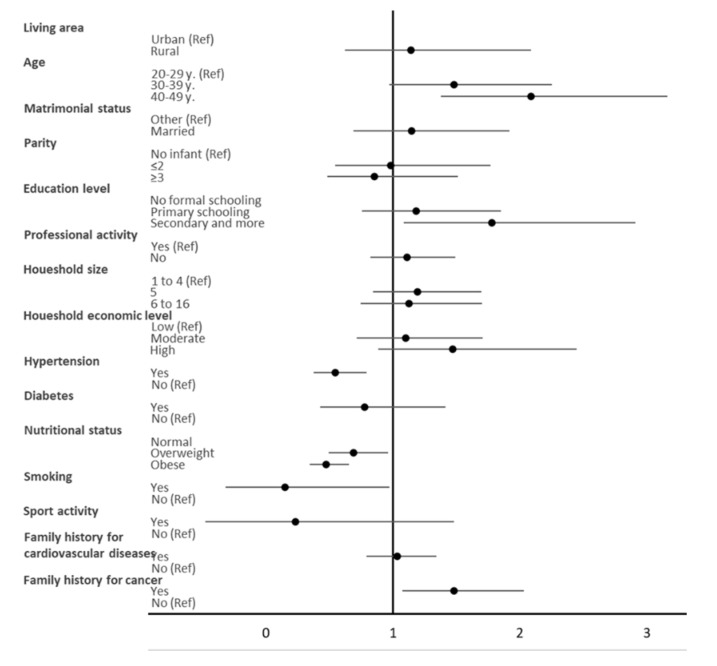
Adjusted odds ratio (OR; 95% CI) of high HDL-C for age (education level, marital status, professional activity, household economic level, household size, and living area), lifestyle (self-reported practice of regular physical activity and smoking), and biological factors (hypertension status, diabetes status, family history of cancer, and family history of cardiovascular diseases).

The results of the multivariate regression analysis (Figure 1) revealed that age (40–49 years) (OR = 2.08 (1.37–3.16)), high education level (secondary or more), hypertension (OR = 0.54 (0.37–0.79)), smoking (OR = 0.56 (0.32–0.97)), and family history of cancer (OR = 1.48 (1.08–2.03)) were the only factors correlated with HDL-C levels in Tunisian women of childbearing age.

## 4. Discussion

The mean HDL-C level found in this study (52.6 ± 0.8 mg/dL) was in the normal range (between 50 and 60 mg/dL) [19] and similar to that reported in previous Tunisian research on dyslipidemia, conducted among 1484 women aged 35–70 years old, in the same sampling area [25]. Compared to data registered elsewhere, the mean HDL-C value in Tunisian women of childbearing age was higher than that recorded in Japanese [26], Korean [27], Hispanic, and African American women [28], and lower than that reported in Canadian [29], Danish [14], and US women [30]. The differences in HDL-C levels between various races and ethnic groups may in part be due to genetic factors, but the role of behavioral, environmental, and anthropometric covariates seems to be important too [31,32].

Age appears to be an independent negative risk factor that can affect HDL-C levels in Tunisian women. This is consistent with previous studies reporting a decrease of HDL-C with age in women [27,33]. Many factors could explain this phenomenon, such as the frequency of insulin resistance and impaired lipolysis at an advanced age that could affect the RCT. Inflammatory processes in aged people, as well as hormonal changes, are other possible causes of the decline in HDL-C with age [34].

A high level of education was found to increase the odds of high HDL-C. This association could be explained by the fact that women who have a higher degree of education might have a better lifestyle, namely practicing regular physical activity, having better knowledge of a healthy diet, and being less stressed by economic hardships [35,36]. These factors are determinants of the cardio-metabolic health and therefore of HDL-C [37].

Parity and marital status negatively influenced the HDL-C concentration in Tunisian women. After pregnancy, the level of cholesterol bound to HDL particles tends to decrease, which explains the tendency of multiparous women to have lower circulating HDL-C levels than women who have never given birth. These changes in circulating cholesterol levels are likely due to changes in estrogen levels, which vary throughout a woman’s genital life [38,39].

Menopause did not affect circulating HDL-C levels. This result is in contradiction with those of several authors showing that a worse lipid profile is observed in postmenopausal women in comparison to premenopausal ones due to hormonal changes involving the decrease in estrogen level and increase in luteinizing hormone and follicle-stimulating hormone levels [33,40]. In our study, the majority of women (92.3%) were premenopausal, which could explain the absence of a relationship between menopause and lipid profile.

In this study, women with high HDL-C levels were more educated and had a lower socioeconomic status than those with average or low levels of HDL-C. Agongo et al. (2018) [41] found a positive significant association between formal education and socioeconomic status with HDL-C levels in women from rural northern Ghana, while no significant association was found between HDL-C and socioeconomic status of Korean women [27]. The mechanisms of association between HDL-C and socioeconomic status are complex due to the influence of lifestyle factors and dietary habits, as well as stress variations by social class [42].

Results on the associations between HDL-C levels and lifestyle factors (physical activity, alcohol consumption, and smoking) showed that smoking was the only negative risk factor of HDL-C in Tunisian women. Research has shown that physical activity and moderate alcohol consumption are positively correlated with HDL-C, contrarily to smoking. According to King et al. (1995), regular physical activity increases the HDL-C level by three to nine percent in healthy sedentary persons [43]. This increase depends on the exercise frequency and intensity, and is attributed to the stimulation of the production of pre-*β* HDL-C and RCT [44]. The effects of smoking on HDL-C are dose-dependent and reversed upon smoking cessation. Nakamura et al. (2020) found that, in both men and women, current smokers had significantly (*p* < 0.001) lower HDL-C than non-smokers (−7.3%, −4.3%) [45]. Likewise, Jain and Ducatman (2018) reported lower HDL-C in smokers than in non-smokers (48.8 vs. 51.4 mg/dL, *p* < 0.01) [46]. Alcohol consumption in moderation raises the concentration of HDL-C, possibly by increasing cellular cholesterol efflux and plasma cholesterol esterification [47]. Brien at al. (2011) reported an increase of HDL-C by 0.1 mmol/L with a quantity of alcohol of about 30 g/day [48]. However, the cardio-protective effect of raised HDL-C by alcohol consumption is largely unknown.

While the univariate analysis showed a higher prevalence of chronic diseases in women with normal or low HDL-C levels (overweight, obesity, abdominal obesity, and hypertension) than the counterpart group, the multivariate regression analysis revealed that hypertension was the only negative risk factor of HDL-C in Tunisian women. Due to epidemiological and nutritional transition, the prevalence of overall obesity and abdominal obesity in Tunisian women has increased drastically during the last few decades [49]. In this study, overall obesity affected a third of Tunisian women, and abdominal obesity concerned almost half, with a decreasing trend with HDL-C levels. The negative associations between obesity and HDL-C have long been reported, and are attributed to the potential role of HDL-C or ApoA-I on adipose tissue content regulation [50,51]. Hypertension is a well-established risk factor for CVDs, and is strongly associated with dyslipidemia, a group of metabolic derangements including low HDL-C levels. This association occurs at the vascular endothelial level, leading to an increase in oxidative stress and endothelial dysfunction [52]. An inverse association between HDL-C and hypertension was reported elsewhere [53]. Halperin et al. (2006) found that men in the highest quintile of HDL-C had a 32% decreased risk of developing hypertension compared with those in the lowest quintile [54]. Likewise, Tohidi et al. (2012) found that women with HDL-C levels between 1.0 and 1.5 mmol L^−1^ had a 33% lower risk of hypertension compared with those who had HDL-C levels < 1 mmol L^−1^ [55].

Family history of chronic diseases (CVDs, hypertension, diabetes, obesity) was not correlated with HDL-C levels in this study, except the family history of cancer. In addition, the intake of lipid-lowering drugs was evenly divided between participants. According to Steyn et al. (1989), women with high levels of HDL-C were less likely to have a history of hypertension or diabetes [56] than those with low HDL-C concentrations. Opoku et al. (2019) reported negative significant associations of the history of coronary heart disease and the history of stroke with HDL-C in Chinese women [53]. In the Bogalusa Heart Study, children with fathers who had a history of myocardial infraction had low ApoA-I levels and a high ApoB/ApoA-I ratio, whereas their HDL-C levels were not outside the normal limits [57]. In this study, the family history of cancer was a strong positive predictor of HDL-C in Tunisian women. Similar findings were observed in a cohort study on US veterans, which reported a slight increase in cancer mortality among participants with high HDL-C levels (>50 mg/dL). However, other epidemiological studies reported that a low HDL-C level may be a risk for cancer deaths or a prognostic factor of many types of cancer in obese subjects [29]. It is worth mentioning that our study did not capture any specific type of cancer, so that the heterogeneity of the types of cancer makes these results hard to interpret. Furthermore, the interpretation might be complicated by a lack of evidence in the association of high cancer risk with genetic forms of hypoalphalipoproteinemia, namely familial LCAT deficiency, familial HDL deficiency due to ABCA1 gene mutations, and familial apoAI deficiency [58]. Women who have faced the loss of a parent due to cancer adopted a better lifestyle, which in turn improved their metabolic health. These controversial results need further investigations on the relationship between HDL-C and cancer.

Significant differences were noticed between biological characteristics in women with high HDL-C levels and the counterpart group. Triglyceridemia, ApoB, TC/HDL-C, ApoB/ApoA-I ratios, SBP, and DBP were lower in women with high HDL-C concentrations, contrary to TC and ApoA-I levels. Increased plasma triglyceride levels have been associated with an increased risk of CVDs, even when the HDL-C levels were adjusted for [59]. ApoA-I is the major structural and functional HDL protein, which accounts for approximately 70% of total HDL protein, and is significantly associated with HDL particles [60]. However, more than 90% of all ApoB in blood is found in LDL [61]. Clinical studies have reported that elevated ApoB levels, an increased apoB/apoA-I ratio, and low levels of apoA-I were better predictors of cardiovascular events than LDL-C, TC, and triglyceride levels, even in patients receiving statins [61]. SBP and DBP were lower in women with high HDL-C levels. This result confirms the protective role of HDL-C against risk factors of CVDs, such as raised blood pressure or hypertension. Despite the significant differences in the biological characteristics between women with high HDL-C and those with normal or low HDL-C, all mean concentrations were within the normal range for both groups in our study.

## 5. Conclusions

The prevalence of high HDL-C and its associated physical, sociodemographic, biological, and lifestyle factors were assessed in a cross-sectional study conducted among Tunisian women of childbearing age. Almost a quarter of the studied women had high HDL-C levels. They were younger, more educated, and had a lower socioeconomic status than those with average or low levels of HDL-C. Age, hypertension, and smoking were independent negative risk factors of high HDL-C in women, while a family history of cancer was positively associated with high HDL-C levels. Due to the controversial findings on the association between high HDL-C and cancer, further investigations should be performed in this domain.

## Figures and Tables

**Table 1:** Characteristics of Tunisian women according to HDL-C levels.

Variable	High HDL-C ≥60 mg/dL (*n* = 446)	Normal and Low HDL-C <60 mg/dL (*n* = 1243)	*p*-Value ^1^
Age (%)			
20–29 years	32.0	28.1	0.35
30–39 years	28.2	31.3	
40–49 years	39.8	40.6	
Area of living (%)			
Rural	6.8	7.5	0.68
Urbain	93.2	92.5	
Marital status (%)			
Other ^2^	61.4	69.2	0.005
Married	38.6	30.8	
Parity (%)			
Three and more children	34.8	42.2	0.029
One or two children	26.6	26.9	
0 children	38.5	30.9	
Menopause (%)			
No	90.5	92.9	0.081
Yes	9.5	7.1	
Level of education (%)			
No schooling	7.7	12.0	<10^−4^
Primary and secondary school	28.3	38.8	
Secondary complete and graduate	64.0	49.2	
Professional activity (%)			
No	33.9	32.4	0.67
Yes	66.1	67.6	
Economic level (%)			
Low	42.5	35.1	0.007
Medium	31.3	34.9	
High	26.2	30.0	
Smoking (%)			
No	94.8	93.6	0.21
Yes	5.2	6.4	
Drinking alcohol (%)			
No	100	99.4	0.24
Yes	0	0.6	
Sport activity (%)			
No	93.2	93.8	0.70
Yes	6.8	6.2	
Hypertension (%)	4.0	6.4	0.074
Diabetes Mellitus (%)	13.1	24.4	<10^−4^
Metabolic syndrome (%)	28.3	33.2	0.059
Family history of cancer (%)	61.2	71.8	0.002
Family history of CVD ^3^ (%)	61.9	66.1	0.17
Family history of hypertension (%)	62.1	64.4	0.59
Family history of diabetes (%)	60.0	57.7	0.48
Family history of obesity (%)	55.2	54.6	0.86
Lipid lowering treatment (%)	1.3	1.3	0.97
Fasting blood glucose (mmol/L)	4.93 ± 0.08	5.07 ± 0.06	0.102
TC ^4^ (mmol/L)	5.17 ± 0.06	4.62 ± 0.05	<10^−4^
Triglyceridemia (mmol/L)	0.89 ± 0.03	1.12 ± 0.03	<10^−4^
LDL-C ^5^ (mmol/L)	2.98 ± 0.06	2.91 ± 0.04	0.27
TC/HDL-C ^6^	2.92 ± 0.04	3.91 ± 0.04	<10^−4^
ApoA-I ^7^ (mmol/L)	1.58 ± 0.03	1.31 ± 0.02	<10^−4^
ApoB ^8^ (mmol/L)	0.79 ± 0.02	0.86 ± 0.02	0.003
ApoB/Apo AI	0.50 ± 0.03	0.66 ± 0.02	<10^−4^
SBP ^9^	119.5 ± 0.9	122.8 ± 0.8	0.001
DBP ^10^	74.5 ± 0.5	76.85 ± 0.51	10^−4^

^1^*p*-Value for logistic regression models accounting for survey design among categories of variable. ^2^ Single, divorced or widowed. ^3^ CVD: cardiovascular diseases. ^4^ TC: total cholesterol. ^5^ LDL-C: low-density lipoprotein cholesterolemia. ^6^ TC/HDL-C: total cholesterol / high-density lipoprotein cholesterolemia. ^7^ ApoA-I: apoliprotein A-I. ^8^ ApoB: apoliprotein B. ^9^ SBP: systolic blood pressure. ^10^ DBP: diastolic blood pressure.

## Data Availability

The data presented in this study are available upon request from the corresponding author.
